# Is the Reluctance for the Implantation of Right Kidneys Justified: Reply

**DOI:** 10.1007/s00268-016-3808-3

**Published:** 2016-11-16

**Authors:** Denise M. D. Özdemir-van Brunschot, Michiel C. Warlé

**Affiliations:** 1 Department of Vascular Surgery, Augusta Krankenhaus, Amalienstraße 9, 40472 Düsseldorf, Germany; 2 Department of Vascular and Transplant Surgery, Radboud University Medical Center, Nijmegen, The Netherlands

Dear Editor,

We thank Khan et al. for their valuable comments to our paper entitled “Is the reluctance for the implantation of right kidneys justified?” [1].

We fully agree with their statement that a short and thin-walled right renal vein can make the venous anastomosis challenging especially in living donor kidney transplantation. In a recent prospective cohort study, we demonstrated that the renal vein length after laparoscopic donor nephrectomy (LDN) could be predicted by preoperative computed tomography (CT) [2]. It was also shown that probably due to the use of the endostapler the ex vivo renal vein length was 11 mm shorter as compared to the preoperative CT measurements. Therefore, we agree with the authors that, at least in theory, open donor nephrectomy (ODN) for retrieving right kidneys may help to overcome the disadvantages of a short renal vein in selected cases. However, we still advocate laparoscopic procurement as the gold standard approach as several high-quality randomized trials showed that LDN is associated with reduced analgesia consumption, shorter length of hospital stay and faster return to normal physical functioning as compared to ODN [3].

Unfortunately, the reasons for discarding 160 right kidneys in a cohort of 2753 deceased kidney donors were not included in the Dutch Organ Transplant Registry (NOTR). Therefore, every explanation remains speculative. Nevertheless, we believe that left deceased donor kidneys are often preferred over their right counterparts during the allocation process. Despite the presence of a caval patch that could be used to augment the right renal vein, the presence of a (very) short right renal vein might be an additional reason to discard “marginal” deceased donor kidneys.

We argued that in case of a perirenal hematoma or urinary obstruction with distension of the pyelum, vascular problems might arise especially if there is a short renal vein with a long renal artery. We agree with Khan et al. that a mismatch in length of the renal vein and artery is particularly associated with a higher risk of arterial kinking. Therefore, it would indeed be advisable to shorten relatively long renal arteries to match the length of the renal vein.

Our retrospective analysis of a large cohort of kidney transplant recipients does not provide evidence for a causal relationship between the procurement of right kidneys and poorer long-term outcome, i.e., long-term graft survival. We agree with Khan et al. that a longer second warm ischemia time not necessarily leads to an increased incidence of delayed graft function. As many variables influence long-term graft survival, we confined our conclusions to the primary outcome measure, i.e., technique-related graft failure. After correction for possible confounders in a multivariable analysis, we have shown that the implantation of right kidneys from living donors is associated with a higher incidence of technical graft failure.
